# *Bucephalus damriyasai* n. sp. (Digenea: Bucephalidae) from the blacktip trevally *Caranx heberi* (Bennett) (Perciformes: Carangidae) off Bali, Indonesia

**DOI:** 10.1007/s11230-018-9828-7

**Published:** 2018-11-21

**Authors:** Rodney A. Bray, Harry W. Palm, Stefan Theisen

**Affiliations:** 1 Department of Life Sciences, Natural History Museum, Cromwell Road, London, SW7 5BD UK; 2 Faculty of Agricultural and Environmental Sciences, Aquaculture and Sea-Ranching, University of Rostock, Justus-von-Liebig-Weg 6, 18059 Rostock, Germany; 3 Faculty of Veterinary Sciences, Udayana University, Bukit Jimbaran, 80363 Badung, Bali Indonesia

## Abstract

The new species *Bucephalus damriyasai* n. sp. is described from *Caranx heberi* (Bennett) from off Bali, Indonesia. It can be distinguished from other *Bucephalus* spp. recorded from carangid hosts by its narrow elongate body shape and the relatively long distance between the rhynchus and the vitellarium, as well as other features distinguishing it from individual species. The most similar species are differentiated from *B. damriyasai* n. sp. as follows: *B. carangis* Yamaguti, 1970 has a much greater length, the rhynchus is smaller and the cirrus-sac is small, not always reaching to the posterior testis; *B. fragilis* Velasquez, 1959 is a tiny species, the pre-vitelline distance is short and the caecum is saccular; *B. gorgon* (Linton, 1905) is much longer and relatively broader, the uterus reaches distinctly anterior to the vitellarium and the rhyncheal tentacles appear more complex; *B. labracis* Paggi & Orecchia, 1965 is distinctly longer, slightly broader, with a slightly larger rhynchus, and has shorter pre-uterine and pre-mouth distances; *B. paraheterotentaculatus* Velasquez, 1959 is much longer, relatively rather broad, the rhynchus is said to bear 21 tentacles, the post-testicular region and cirrus-sac reach are longer and the caecum is described as saccular; *B. sphyraenae* Yamaguti, 1952 is longer, slightly broader, the uterus reaches anteriorly to the vitellarium and the caecum is claviform and oriented anteriorly; *B. margaritae* Ozaki & Ishibashi, 1934 (syn. *B. varicus* Manter, 1940) is relatively squat, has shorter pre-vitelline and pre-mouth distances and a longer post-testicular distance and cirrus-sac reach; *B. yamagutii* Gupta & Singh, 1985 is broader, with a relatively short pre-vitelline distance, the caecum extends anteriorly to the pharynx, but not posteriorly and the rhynchus is said to carry five tentacles. The distinctive features of *B. damriyasai* n. sp. are compared with those of all other marine *Bucephalus* spp. in a table. The number of bucephalid trematodes known from Indonesian waters is now 13, two of them await further identification. They have been described from the fish families Carangidae, Platycephalidae, Sciaenidae, Serranidae and Sphyraenidae.

## Introduction

The Indonesian fish parasite fauna is species-rich, resulting from the high number of fish species surrounding about 17,000 islands of this maritime nation. However, though being a hot spot of aquatic biodiversity, fish parasites are far less studied. As stated by Bray & Palm (2009) and considering recent studies (Bray et al., 2016; Yong et al., 2016; Bray et al., 2017), over 80 fish-parasitic trematode species have been reported from Indonesian waters.

According to Palm & Bray (2014), many fish parasites that have been reported off the Hawaiian Islands in the Central Pacific have an Indo-Pacific or even worldwide distribution. Although it can be expected that a high number of new trematode taxa will be described from Indonesia, the same importance should be placed on having reliable identifications of already known species from other regions. Without proper identification the use of fish parasites as biological indicators (Palm, 2011), of increasing importance in many regions (Truong et al., 2017), is difficult and in some cases impossible. Palm & Rückert (2009), Palm et al. (2011) and Neubert et al. (2016) have developed a method to use grouper fish parasites as biological indicators for pollution and environmental change in Indonesian coastal waters, but several bucephalids recovered were only tentatively identified (Bray & Palm, 2009).

The genus *Bucephalus* Baer, 1826 includes many species reported from fresh and marine waters. It is characterised by having a sucker-like rhynchus with a hood bearing tentacles (usually seven). The tentacles may be found withdrawn and difficult to see and in such cases the worms look very similar to members of *Rhipidocotyle* Diesing, 1858. Recent molecular studies by Nolan et al. (2015) have indicated that the genus is polyphyletic, with at least three separate monophyletic groups embedded within an assortment of species of *Rhipidocotyle*, *Prosorhynchoides* Dollfus, 1929 and *Paurorhynchus* Dickerman, 1954.

*Caranx heberi* (Bennett) (syn. *C. sem* Cuvier) is a common carangid throughout the Indo-Pacific region. The only reports of digeneans we are aware of from this host are the bucephalid *Bucephalus margaritae* Ozaki & Ishibashi 1934 from off Natal, South Africa (Bray, 1984) and an unidentified sclerodistomid *Prosorchis* sp. from the Arabian Gulf (El-Naffar et al., 1992; Al Kawari et al., 1996). We herewith present a description of new bucephalid species from this host from the Balinese coast, Indonesia.

## Materials and methods

The present study is based on material collected during the First Educational Workshop on Marine Fish Parasites in Bali, July 21st - August 2nd 2013, of Indonesian and international students and researchers investigating a wide range of hosts from Balinese waters. Three specimens (15.7–16.7 cm total length, 66.2–70.6 g) of *Caranx heberi* were caught by artisanal fishermen and landed at Kedonganan Bay, transported alive into the laboratory of the Veterinary Faculty, Udayana (UNUD) University, Denpasar, Bali, and directly studied for fish parasites. Kedonganan Bay is located at the western side of the southern tip of Bali, directly next to the Ngurah Rai Kuta international airport. The airstrip of the airport reaches into the ocean and acts as the northern border of the bay. Kedonganan is a typical fishing village but is heavily influenced by tourism. There is no harbour; the small ships lay directly in front of the beach which is used to land captures. The fishermen catch fish from the Bali Strait and from nearby areas off South Bali and East Java. They use drift nets, troll lines and hand lines. A cooperation of the local fishermen manages the market (Proctor et al., 2003). Digeneans were collected according to the gut wash methodology described by Cribb & Bray (2010). Unfortunately, although these worms were collected with a view to sequencing, these specimens have not yielded usable rDNA.

Whole-mounts were stained with Mayer’s paracarmine, cleared in beechwood creosote and mounted in Canada balsam. Measurements were made through a drawing tube on an Olympus BH-2 microscope, using a Digicad Plus digitising tablet and Carl Zeiss KS100 software adapted by Imaging Associates, and are quoted in micrometres, as the range and the mean in parentheses. ‘Cirrus-sac reach’ is the distance between the posterior extremity of the worm and the anteriormost extent of the cirrus-sac. The type-material is deposited in the following museum collections: the Natural History Museum, London, UK (NHMUK); the National Biodiversity Collection, Museum Zoologicum Bogoriense, Cibinong, Bogor, Java, Indonesia (MZB); and the Natural History Museum, Berlin, Germany (ZMB).

## Results

All *C. heberi* sampled were infected with a new bucephalid (100% prevalence), but levels of intensity were not recorded.

**Family Bucephalidae Poche, 1907**

**Genus** ***Bucephalus*** **Baer, 1827**

***Bucephalus damriyasai*** **n. sp.**

*Type-host*: *Caranx heberi* (Bennett) (Perciformes: Carangidae), blacktip trevally.

*Type-locality*: Off South Bali, Indonesia. Purchased from artisanal fishermen, 23.vii.2013, 26.vii.2013, 31.vii.2013.

*Type-specimens*: Holotype ZMB E.7629. Paratypes: E.7630–1; MZBTr 246–250; NHMUK 2018.6.7.1–3.

Site in host: Intestine.

*ZooBank registration*: To comply with the regulations set out in article 8.5 of the amended 2012 version of the *International Code of Zoological Nomenclature* (ICZN, 2012), details of the new species have been submitted to ZooBank. The Life Science Identifier (LSID) for *Bucephalus damriyasai* n. sp. is urn:lsid:zoobank.org:act:F68217B4-831B-4D2A-84CE-F731F41CFCFC.

*Etymology*: This species is named in honour of Professor Dr I Made Damriyasa, Faculty of Veterinary Sciences, Udayana University, for his continuous support of fish parasite research in Balinese waters.

### Description (Figs. 1, 2)

[Based on 15 whole-mount preparations.] Body elongate, narrow, gradually widening to maximum at about level of gonads; length 851–1,544 × 76–123 (1,126 × 97); width 7.57–10.4 (8.71)% of body length. Tegument spinous throughout; spines tiny. Rhynchus 67–85 × 55–87 (77 × 71), bearing 7 tentacles, each with side branches and elongate central branch; most often withdrawn and difficult to see; rhynchus length 5.53–8.34 (7.01)% of body length; rhynchus width 77.8–112 (92.7)% of rhynchus length. Mouth at level of ovary, well inside posterior half of body, pre-mouth distance 559–1,011 (737), 63.4–69.1 (65.4)% of body length. Pharynx globular; 48–66 × 47–60 (59 × 55), width 3.82–6.32 (5.00)% of body length. Caecum elongate, 72–450 × 16–44 (351 × 27); length 28.1–44.3 (33.8)% of body length; mostly reaching anterior to pharynx almost to anterior extremity of vitellarium, but with distinct posteriorly directed part overlapping testes, pre-caecal distance 234–706 (452), 27.5–52.6 (38.8)% of body length; caecum to rhynchus distance 164–614 (385).

**Figs. 1–2:**
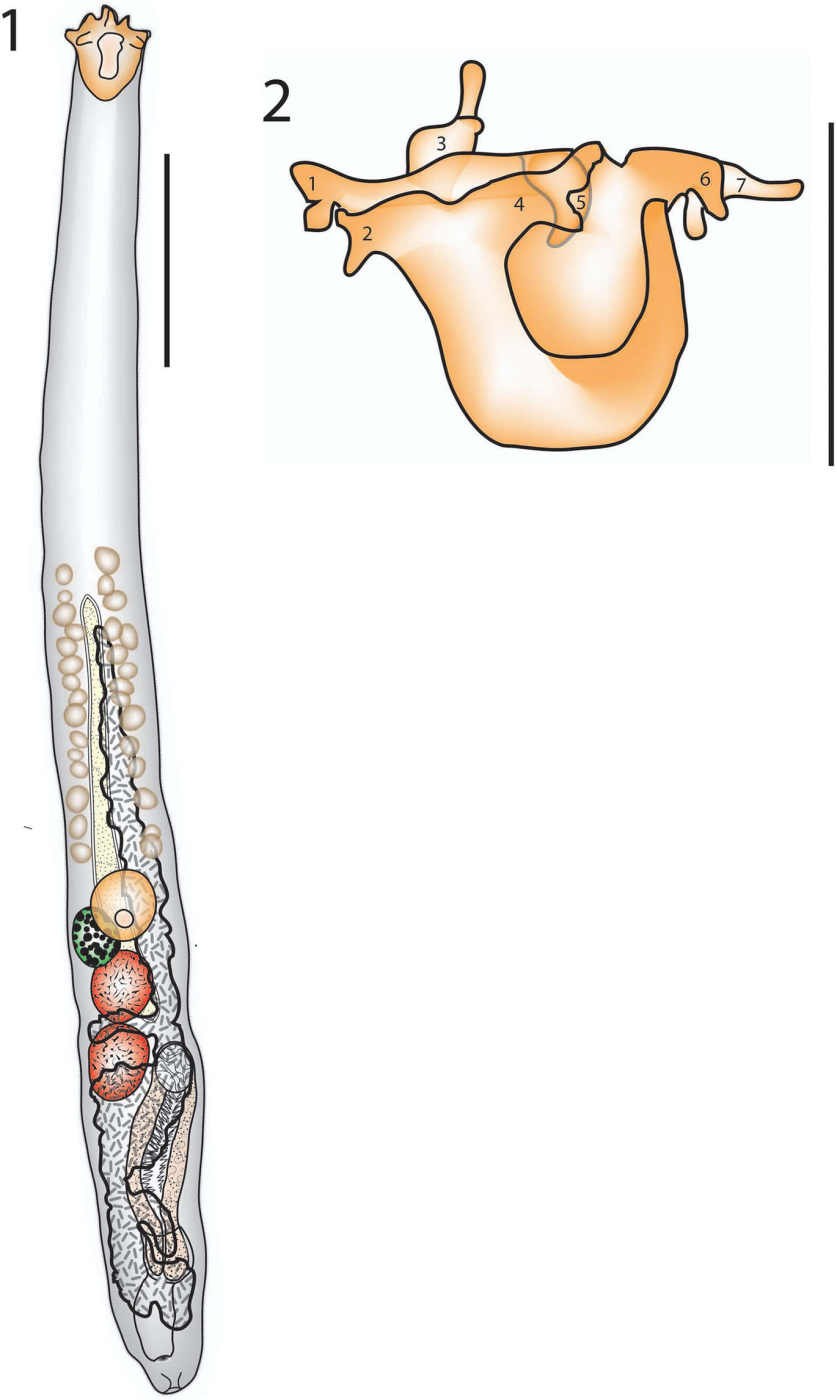
*Bucephalus damriyasai* n. sp. 1, Holotype, ventral view; 2, Rhynchus with the seven tentacles labelled for clarity. *Scale-bars*: 1, 200 µm; 2, 100 µm

Testes 2, oval, tandem or nearly so, in posterior quarter of body; pre-testicular distance 573–1,092 (767), 65.0–71.2 (68.0)% of body length; contiguous or slightly separated, distance 0–19 (8), 0–1.99 (0.78)% of body length; anterior testis 44–78 × 42–69 (58 × 53), length 4.74–5.70 (5.11)% of body length; posterior testis 39–86 × 39–61 (60 × 52), length 4.56–6.47 (5.27)% of body length; post-testicular distance 160–283 (216), 16.9–22.2 (19.3)% of body length. Cirrus-sac elongate, more or less parallel sided, reaching to posterior or anterior testis; 125–254 × 33–58 (172 × 43), length 13.2–16.9 (15.3)% of body length; cirrus-sac reach 208–359 (276), 21.7–28.1 (24.7)% of body length. Seminal vesicle subglobular to oval, in proximal cirrus-sac; 26–52 × 23–41 (37 × 32), length 18.9–25.2 (21.4)% of cirrus-sac length. Pars prostatica long, straight, surrounded by dense layer of gland-cells, lined with filaments; 91–195 (127) long, 20–29 (25) wide. Ejaculatory duct narrow, opens on large, lobed genital lobe, inside genital atrium. Genital atrium large. Genital pore ventral, distinctly separated from posterior extremity by 23–42 (34), 2.46–4.16 (3.07)% of body length.

Ovary oval, 41–75 × 35–62 (55 × 45), length 4.19–6.09 (4.84)% of body length; pre-testicular, contiguous with anterior testis; pre-ovarian distance 543–1,030 (724), 61.4–66.7 (64.2)% of body length; post-ovarian distance 245–457 (332), 26.7–32.0 (29.6)% of body length. Mehlis’ gland overlaps ovary and anterior testis. Uterine seminal receptacle and Laurer’s canal not detected. Uterus not usually reaching anteriorly to vitelline fields, pre-uterine distance 322–853 (514), 33.1–55.2 (45.0)% of body length; uterus to rhynchus distance 254–771 (447), uterus narrow anteriorly to pharynx, wider posteriorly. Eggs numerous, tanned, operculate; 18–25 × 13–19 (22 × 16). Metraterm not detected, obscured by eggs. Vitellarium consists of 2 lateral fields of 14–18 (16) follicles, symmetrical or nearly so, but with one slightly longer than other, long field 180–385 (265) long, 20.3–26.5 (23.3)% of body length; shorter field 168–360 (248) long; anterior extremity usually anterior to uterus and caecum; pre-vitelline distance 359–587 (438), 36.9–42.4 (39.0)% of body length; vitellarium to rhynchus distance 287–504 (363); posterior extremity just anterior to or overlapping pharynx; post-vitelline distance 290–600 (415), 33.5–39.7 (36.9)% of body length.

Excretory pore terminal; anterior extent of vesicle visible in some specimens reaching just anterior to vitellarium.

## Discussion

We are aware of 48 described nominal species of *Bucephalus* in marine fishes and we have examined the descriptions of all except *B. arabiana* Varma, 1982, the description of which we have been unable to find. We have developed a visual key similar to that to *Prosorhynchus* developed by Bray & Palm (2009) (http://www.nhm.ac.uk/bray2009). Ten characters are used, most of which are listed as a percentage of body length: 1, Length; 2, Width %; 3, Rhynchus length %; 4, Tentacle number; 5, Pre-vitelline distance %; 6, Pre-uterine distance %; 7, Pre-mouth distance %; 8, Post-testicular distance %; 9, Cirrus-sac reach %; and 10, Egg length.

Eight species have none of the above listed percentage characters more than 10% either more or less than that quoted in the original description or derived from the original illustrations, or are distinctly different in size, tentacle number or egg-size. These are *B. carangis* Yamaguti, 1970; *B. fragilis* Velasquez, 1959; *B. gorgon* (Linton, 1905) (syn. *B. introversus* Manter, 1940); *B. labracis* Paggi & Orecchia, 1965; *B. paraheterotentaculatus* Velasquez, 1959; *B. sphyraenae* Yamaguti, 1952; *B. varicus* Manter, 1940 (usually considered a synonym of *B. margaritae* Ozaki & Ishibashi, 1934); and *B. yamagutii* Gupta & Singh, 1985.

*Bucephalus carangis* Yamaguti, 1970 is reported from the black jack *Caranx lugubris* Poey and the bluefin trevally *C. melampygus* Cuvier off Hawaii (Yamaguti, 1970; Palm & Bray, 2014). It apparently grows to a much greater size than *B. damriyasai* n. sp. (to 3,500 µm), the rhynchus is smaller (3–5% of body length), and the cirrus-sac is small, not always reaching to the posterior testis (cirrus-sac reach about 18% of body length).

*Bucephalus fragilis* Velasquez, 1959 is also reported from carangids, the torpedo scad *Megalaspis cordyla* (L.), the doublespotted queenfish *Scomberoides lysan* (Forsskål) and *Caranx* sp. from off the Philippines, the South China Sea and Masirah Bay off Oman in the northern Indian Ocean (Velasquez, 1959; Parukhin, 1966, 1976). In this tiny species (length 660–900 µm), the pre-vitelline distance is short (about 27% of body-length) and the caecum is saccular.

*Bucephalus gorgon* (Linton, 1905) (syn. *Bucephalus introversus* Manter, 1940) is a widely reported species known only from carangids and mainly from members of the genus *Seriola* in the Pacific, Indian and Atlantic Oceans. It was described by Linton (1905) as *Gasterostomum gorgon* in the yellowtail amberjack *Seriola lalandi* Valenciennes from the North-West Atlantic at Beaufort, North Carolina. Eckmann (1932) placed this species in *Bucephalus*. It was redescribed and illustrated (apparently a badly contracted specimen) by Linton (1940) as *Nannoenterum gorgon* from *S. lalandi* at Woods Hole, Massachusetts. Bartoli et al. (2005) redescribed this species from the greater amberjack *Seriola dumerili* (Risso) off Corsica in the western Mediterranean Sea. They considered *Bucephalus introversus* Manter, 1940, from the crevalle jack *Caranx hippos* (L.)*, S. dumerili*, *S. lalandi* and *Seriola* sp. from the eastern Pacific off Mexico and Columbia (Manter, 1940a) as a synonym of *B. gorgon*. Further descriptions and descriptive matter have been given by Oshmarin (1965) from *Seriola* ‘*nigromaculata*’ off Vietnam; Corkum (1967) from *S. dumerili*, and the banded rudderfish *Seriola zonata* (Mitchill) off Louisiana; Fischthal et al. (1982) from *S. dumerili* off Israel in the eastern Mediterranean; and Luque & Oliva (1993) from *S. lalandi* (as *S. mazatlana*) off Antofagasta, Chile. Other hosts recorded are the threadfin jack, *Carangoides otrynter* (Jordan & Gilbert), the white trevally *Pseudocaranx dentex* (Bloch & Schneider) and the Almaco jack *Seriola rivoliana* Cuvier and the distribution includes the Gulf of Mexico and off Canary Islands in the Atlantic, the Balearic Sea in the western Mediterranean, the Gulf of Mannar in the Indian Ocean and the South China Sea and off New South Wales and Victoria, eastern Australia in the Pacific Ocean (Bravo-Hollis & Sogandares-Bernal, 1956; Parukhin, 1966; Nahhas & Powell, 1971; Parukhin, 1976; Fischthal, 1982; Montero et al., 2002; Gijon-Botella et al., 2007; Hutson et al., 2007). Nolan et al. (2015) used sequences of *B. gorgon* from *S. dumerili* in the Gulf of Mexico to show that the closest sequenced relative is *Prosorhynchoides ovatus* (Linton, 1898).

Linton (1905, p. 364) described the “anterior sucker (i.e. a rhynchus) surrounded by a crown of about eighteen tentacles”; in most cases, the anterior end of his specimens was withdrawn (his figure 241; incorrectly oriented). Linton (1940) redescribed the species with an anterior sucker provided with about 20 tentacles, the specimens being “all macerated”. Bartoli et al. (2005) stated that “the rhynchus of *B. gorgon* consists of seven large retractile tentacles, each of them provided with one or two small basal processes”. *Bucephalus gorgon* is usually described as much longer and relatively broader than *B. damriyasai* n. sp., the uterus reaches distinctly anterior to the vitellarium. The rhyncheal tentacles appear much more complex in *B. gorgon*, particularly as described by Bartoli et al. (2005).

*Bucephalus labracis* Paggi & Orecchia, 1965 was originally found in the European seabass, *Dicentrarchus labrax* (L.) in the Tyrrhenian Sea (Paggi & Orecchia, 1965). It has subsequently been reported from the same host off Israel, off the Iberian Peninsula, in the Tunisian and Algerian lagoons and off Sardinia (Fischthal, 1982; Muñoz et al., 1989; Gargouri Ben Abdallah & Maamouri, 2005; Gijon-Botella et al., 2007; Culurgioni et al., 2010; Culurgioni et al., 2014; Brahim Tazi et al., 2016). This species grows to 3,310 µm long, is slightly or distinctly broader with a slightly larger rhynchus, has shorter pre-uterine (about 23–29%) and pre-mouth (about 54–57%) distances. Metacercariae have been reported in the big-scale sand smelt *Atherina boyeri* Risso, the common goby *Pomatoschistus microps* (Krøyer), the black-striped pipefish *Syngnathus abaster* Risso, the leaping mullet *Chelon saliens* (Risso), the golden grey mullet, *C. auratus* (Risso) and the gilthead seabream *Sparus aurata* L. (see Gargouri Ben Abdallah & Maamouri, 2005; Culurgioni et al., 2014; Culurgioni et al., 2015). The first intermediate host is the carpet shell *Tapes decussatus* (L.) (Gargouri Ben Abdallah & Maamouri, 2005). Another species has been described under this name, *Bucephalus labracis* Nisreen Ezz El-Dien, Abdel-Rahman, El-Gawady, Imam & Fahmy, 1990, from the same host species in the Suez Canal at Ismailia, Egypt (Nisreen Ezz El-Dien et al., 1990). The description is poor, but it does not appear to be conspecific with its senior homonym and may not be in this genus.

*Bucephalus paraheterotentaculatus* Velasquez, 1959 was originally reported in the blackbanded trevally *Seriolina* [as *Seriola*] *nigrofasciata* (Rüppell) from Malabon, Rizai, Luzon Island, Philippines (Velasquez, 1959). It has subsequently been reported in *S. nigrofasciata*, *S. dumerili* and *Seriola* sp. from the South China Sea and Masirah Bay off Oman in the Northern Indian Ocean (Parukhin, 1966, 1976). This species grows to 4,070 µm long, is relatively rather broad (maximum width about 12–13% of length), the rhynchus bears “21 tentacles grouped in multiples of 3 conforming to the basic number of 7”, the post-testicular region is about 31% of body length and the cirrus-sac reach is about 36% of body length. The caecum is described as saccular.

*Bucephalus sphyraenae* Yamaguti, 1952 was originally reported from *Sphyraena* sp. off Makassar, Sulawasi, Indonesia (Yamaguti, 1952). Subsequently it has been reported from the blackfin barracuda *Sphyraena qenie* Klunzinger (as *S. tessera*), the false stonefish *Scorpaenopsis diabolus* (Cuvier), the obtuse barracuda, *Sphyraena obtusata* Cuvier and the yellowstripe barracuda *Sphyraena chrysotaenia* Klunzinger from the Red Sea, off Okinawa, Japan and the Arabian Gulf off Kuwait (Parukhin, 1970; Dyer et al., 1988; Nahhas et al., 2006). This species grows to 2,800 µm, its width is about 12–16% of body length and the uterus reaches anteriorly to the vitellarium. The caecum is claviform and oriented anteriorly.

*Bucephalus varicus* Manter, 1940 is usually considered one of several synonyms of *B. margaritae* Ozaki & Ishibashi, 1934. A fairly high proportion of records of *Bucephalus* species from carangids are of *B. margaritae* and its synonyms. It was originally described as a furcocercous cercaria from the pearl oyster *Pinctada imbricata* Röding (as *Pinctada imbricata mertensii*) off Japan (Ozaki & Ishibashi, 1934). A series of papers by Sakaguchi (1962, 1964, 1966a, b, 1968) reported on the completion of the life-cycle of this species and concluded that it was conspecific with *B. varicus* Manter, 1940. Manter (1940a) in describing *B. varicus* from “a young specimen of an unidentified species of *Caranx*, or jack” off Bahia Honda, Panama, considered that *B. polymorphus* of Nagaty (1937) from carangids in the Red Sea was a misidentification, as *B. polymorphus* is a freshwater species. Overstreet (1969) and Velasquez (1975) considered *B. pseudovaricus* Velasquez, 1959 synonymous with *B. varicus.* Bray (1984) also considered that *B. retractilis* Yamaguti, 1959, *B. carangoides* Yamaguti, 1970 and *B. ulua* Yamaguti, 1970 are synonyms of *B. margaritae*. Nahhas et al. (2006) “confirm this synonymy” and described the worm from the cleftbelly trevally *Atropus atropos* (Bloch & Schneider), the largemouth queenfish *Scomberoides commersonnianus* Lacépède, the Malabar trevally *Carangoides malabaricus* (Bloch & Schneider), the whipfin silver-biddy *Gerres filamentosus* Cuvier and the pickhandle barracuda *Sphyraena jello* Cuvier in the Arabian Gulf off Kuwait. Chinchilla et al. (2006) accepted these synonymies, and described the worm from the southern sennet *Sphyraena picudilla* Poey off Venezuela. Marchiori et al. (2010) also accepted the synonymy and described the life-cycle in the brown or South American rock mussel *Perna perna* (L.), the combtooth blenny *Hypleurochilus fissicornis* (Quoy & Gaimard) and the southern kingcroaker *Menticirrhus americanus* (L.) in the waters off Brazil. Many of the more recent records of *B. margaritae* are of non-carangid hosts. Al-Zubaidy (2011) described *B. margaritae* and *B. varicus* as separate species, from the great barracuda *Sphyraena barracuda* (Edwards) and the orange-spotted trevally *Carangoides bajad* (Forsskål), respectively, from Yemeni Red Sea coastal waters off Hodeidah. The illustrations suggest that several species are involved. Nolan et al. (2015) used sequences of worms identified as *B. margaritae* from *Caranx crysos* (Mitchill), from the Gulf of Mexico in their molecular study and showed that, of sequenced species, it is the sister of *B. cynoscion* Hopkins, 1956. *Bucephalus margaritae* differs from *B. damriyasai* n. sp. in its relatively squat shape, short pre-vitelline distance, shorter pre-mouth distance and longer post-testicular distance and cirrus-sac reach. The species has been described many times under the same or different names and clearly needs careful revision. It is likely, if not virtually certain, that a complex of similar species is now known under this name.

*Bucephalus yamagutii* Gupta & Singh, 1985 is reported only from the Malabar trevally *Carangoides malabaricus* (Bloch & Schneider) (as *Caranx malabaricus*) off the Puri coast in the Bay of Bengal (Gupta & Singh, 1985). It is relatively broad (width about 16% of length), with a relatively short pre-vitelline distance (about 28% of body-length) and the caecum extends anteriorly to the pharynx, but not posteriorly. The rhynchus is said to carry five tentacles.

The features that distinguish marine *Bucephalus* spp. from *B. damriyasai* n. sp. are tabulated in Table 1.

**Table 1:** Comparative table of marine *Bucephalus* spp. Bold indicates major distinctions, italics indicates minor distinctions. Column 3: Width %; 4: Rhynchus length %; 6: Pre-vitelline distance %; 7: Pre-uterine distance %; 8: Pre-mouth distance %; 9: Post-testicular distance %; 10: Cirrus-sac reach %

	Length	3	4	Tentacle number	6	7	8	9	10	Eggs	Source
*B. damriyasai* n. sp.	851–1,544	8–10	6–8	7	37–42	33–55	63–69	17–22	22–28	18–25 × 13–19	Present study
*B. anguillae* Špakulová, Macko, Berrilli & Dezfuli, 2002	1,118–1,658	**27–29**	*17–18*	7	*21–37*	**16–17**	*54–60*	22–28	34–42	28–30 × 18	Špakulová et al. (2002)
	1,160–2,320	**26–34**	*15–16*	5 (retracted)	*28*	**18**	*57*	15	26	20–34 × 10–18	Gargouri-Ben Abdullah & Maamouri (2002)
*B. arabiansis* Dwivedi, 2007	1,020–1,530	*17–35*	*10–16*	*5*	**12**	**13**	68	23	**44**	20–30 × 10–30	Dwivedi (2007)
*B. baeri* Maillard & Saad-Fares, 1981	1,320–2,640	*15–17*	10–13	7	*32*	**17**	45	22	28	24–27 × 14–16	Maillard & Saad-Fares (1981)
*B. barina* Srivastava, 1938	1,520–2,800	**24–33**	7–9	**5**	**24**	**14**	*55*	**36**	**39**	15–19 × 9–11	Srivastava (1938)
*B. binidentacularis* Wang, 1977	1,600	**35**	*11*	[? 6]	**15**	**13**	*53*	**38**	**46**	21–25 × 14–16	Wang (1977)
*B. brevitentaculatus* Corkum, 1967	660–1,330	**28–40**	9–11	7	**15**	**19**	*54*	**37**	**50**	20 × 13	Corkum (1967)
*B. carangis* Yamaguti, 1970	*1,150–3,500*	10–14	*3–5*	7	34	38	59	22	*18*	17–23 × 11–14	Yamaguti (1970)
*B. carangoides* Yamaguti, 1970	1,000–1,650	**35–44**	*14–15*	7	**21**	**12**	*56*	**39**	**43**	16–21 × 10–14	Yamaguti (1970)
*B. confusus* Velasquez, 1959	**2,800**	12.5	4	**20**	?	?	?	?	?	24 × 13–18	Linton (1940)
*B. cynoscion* Hopkins, 1956	600–1,400	*17–34*	8–16	*5 (or 7)*	**13**	**8**	57	30	32	20–25 × 13–15	Hopkins (1956)
*B. elacatus* Yadav, 1977	**4,160–5,120**	*13–15*	*2–3*	*4*	36	*25*	*53*	18	23	24–25 × 9–11	Yadav (1977)
*B. fischthali* Gupta & Tiwari, 1985	**2,520–4,000**	*18–27*	6–8	*6*	**23**	*25*	63–64	21–23	33	32–35 × 32–35	Gupta & Tiwari (1985)
*B. fragilis* Velasquez, 1959	660–900	*15–26*	6	7	**27**	33	59	27	30	17–18 × 9–13	Velasquez (1959)
*B. gorgon* (Linton, 1905)	1,562–2,750	7–15	8–12	7	31–45	*17–24*	59–65	18–22	26–28	21–24 × 13–15	Bartoli et al. (2005)
	1,650	**22**	11	**about 18**	29	**45**	?	16	34	22 × 14	Linton (1905)
	**2,380–3,130**	11–14	7–10	**about 20**	*28*	*8–30*	?	?	?	18–21 × 10–12	Linton (1940)
	**2,238**	12	9	**22**	33	**17**	59	25	28	?	Corkum (1967)
	**2,700**	9	9	?	*27*	**21**	60	*31*	25	19–20 × 13–14	Oshmarin (1965)
*B. hainanensis* Shen, 1990	1,337–2,278	**27–28**	6–7	7	**19**	**17**	*54*	*30*	*34*	15–18 × 9–12	Shen (1990)
*B. harpodontis* Wang, 1980	1,200–1,600	11	7	8	**22**	34	**44**	26	28	24–26 × 16–18	Wang (1980)
*B. heterotentaculatus* Bravo-Hollis & Sogandares-Bernal, 1956	**2,080–3,420**	12–13	8–11	7	36	**18**	56–78	24	29	22–28 × 13–17	Bravo-Hollis & Sogandares-Bernal (1956)
*B. hexalobatus* Bilqees, Khatoon & Haseeb, 2006^a^	**3,790–3,850**	**21–22**	?	?	?	?	?	?	?	20–24 × 17–19	Bilqees et al. (2006)
*B. introversus* Manter, 1940	1,202–1,707	*17–19*	*14–20*	7	46	30	61	14	*35*	22–26 × 12–15	Manter (1940a)
*B. introversus* Manter, 1940	1,720–2,940	*16*	*3–4*	?	?	?	?	?	?	23 × 17	Luque & Oliva (1993)
*B. jagannathai* Verma, 1936	1,100–1,700	**32–38**	*11–15*	*6*	**21**	**17**	**51**	24	**43**	19–20 × 12–13	Verma (1936)
*B. kaku* Yamaguti, 1970	*1,500–4,700*	11–13	6–7	*11*	*53*	*25*	**48**	19	23	21–25 × 12–16	Yamaguti (1970)
*B. kanagurtai* Gupta & Tiwari, 1985	**3,200–3.570**	**26–27**	7–8	7	**23**	*25*	64–76	*31*	*37*	**40–70 × 40–70 (?)**	Gupta & Tiwari (1985)
*B. kathetostomae* (Manter, 1934)	688–1,434	**20–49**	12–21	*5*	**19**	26	*39–55*	**42**	**53**	18–20 × 10–11	Manter (1934)
*B. labracis* Paggi & Orecchia, 1965	*1,980–3,310*	11–14	*10–11*	7	46	*23*	*54*	16	21	20–21 × 18–19	Paggi & Orecchia (1965)
	*1,370–2,750*	15–18	*10–14*	?	?	?	?	?	?	22–24 × 13–15	Maillard (1976)
	*1,200-2,600*	**22-30**	*13-15*	7	30	29	*57*	18	?	20-38 x 12-23	Gargouri Ben Abdullah & Maamouri (2005)
*B. labracis* Nisreen Ezz El-Dien, Abdel-Rahman, El-Gawady, Imam & Fahmy, 1990	*709–727*	**28–29**	*13–21*	**numerous**	41	45	71	19	**45**	19–24 × 9–10	Nisreen Ezz El-Dien et al. (1990)
*B. leognathi* Velasquez, 1959	*680*	**44**	*13*	*6*	*29*	30	**51**	**39**	*36*	17–18 × 11–13	Velasquez (1959)
*B. margaritae* Ozaki & Ishibashi, 1934	?	*19*	8	7	**21**	**14**	60	25	30	??	Sakaguchi (1968)
	1,435	*15*	9	7	*48*	*29*	59	15	24	20–22,5 × 10	Chinchilla et al. (2006)
	320–815	*16–20*	*15*	7	42	?	67–69	17	*32*	14–28 × 4(?)–19	Marchiori et al. (2010)
	343–834	*17–20*	9	7	42	*28*	*58*	14	25	19–20 × 8–11	Al-Zubaidy (2011)
*B. marinus* Vlasenko, 1931	2,000	10	*12*	7?	33	**21**	**40**	*28*	30	24 × 15	Vlasenko (1931)
*B. minimus* (Stossich, 1887)	900	**72**	*13*	0?	41	44	**35**	**36**	**41**	not given	Stossich (1887)
	840–1,540	**76**	*11–16*	7	**57**	34	**43**	24	**41**	22–24 × 13–14	Maillard (1975, as *Labratrema lamirandi*)
	366–1,088	**51–70**	*10–18*	?	**57**	?	?	?	?	18–24 × 10–15	Pina et al. (2009)
*B. neoscombropsi* Parukhin, 1979	*2,300–2,860*	17	8–9	7	41	27	**50**	16	26	20–25 × 17–22	Parukhin (1979)
*B. paraheterotentaculatus* Velasquez, 1959	*1,220–4,070*	12–13	*2–3*	21/3 = 7	40	31	42–71	*31*	*36*	15–26 × 11–18	Velasquez (1959)
*B. priacanthi* Manter, 1940	1,020–1,215	**21**	*12*	7	**25**	**13**	**51–52**	24	**39**	17–19 × 10–12	Manter (1940b)
*B. pseudovaricus* Velasquez, 1959	980–1,000	**24–30**	8	weak	36	34	59	24	**43**	18–22 × 13–14	Velasquez (1959)
*B. retractilis* Yamaguti, 1952	1,400–1,950	**21–23**	7	7	**18**	**16**	*55*	**35**	*37*	15–16 × 10–12	Yamaguti (1952)
*B. scorpaenae* Manter, 1940	*2,065–2,792*	*12–15*	6–7	7	**23**	**15**	**46**	23	28	19–22 × 13–15	Manter (1940b)
*B. sebastichthydis* Yamaguti, 1959	**3,450**	*13*	10	7	*31*	*25*	**51**	22	23	23–26 × 13–16	Yamaguti (1959)
*B. sextentaculatus* Yamaguti, 1970	*1,800–3,600*	8–9	8	*6*	38	**7**	63	26	21	16–21 × 11–16	Yamaguti (1970)
*B. solitarius* Kohn, 1966	1,550	*15*	7	*5*	33	**21**	60	*32*	28	20–22 × 11–13	Kohn (1966)
*B. sphyraenae* Yamaguti, 1952	*2,500–2,800*	*12–16*	*5*	7	37	*25*	*58*	25	25	18–24 × 12–16	Yamaguti (1952)
	*1,500–2,175*	*12–15*	*5–6*	7	*29*	**16**	**46**	21	26	13–20 × 10–18	Nahhas et al. (2006)
*B. thapari* Gupta & Tiwari, 1983	**3,470–3,850**	**25–26**	7–8	*4*	*29*	*29*	68	*28–30*	**39**	16–18 × 16–18	Gupta & Tiwari (1985)
*B. trifurcatus* Wang, 1980	*2,400–2,480*	10	6	7	**23**	**17**	55	21	21	19–21 × 14–15	Wang (1980)
*B. ulua* Yamaguti, 1970	650–1,300	**28–43**	*14–15*	7 (rarely 6)	**21**	**19**	63	**35**	**47**	14–21 × 9–14	Yamaguti (1970)
*B. uranoscopi* Yamaguti, 1934	**4,860**	9	*3*	7	40	41	63	22	*19*	18 × 22	Yamaguti (1934)
*B. urophyci* Szidat, 1961	760	*13*	*17*	?	*28*	**20**	*55*	25	*37*	19 × 10	Szidat (1961)
*B. varicus* Manter, 1940	705–1,458	*9–28*	*6–19*	7	**20–25**	*17–31*	*53*	21–49	*35–48*	17–20 × 9–16	Manter (1940a)
	618–2,288	*15–17*	8–14	7	23–38	*25–30*	48–86	22–31	*32–54*	21–27 × 13–23	Nagaty (1937, as *B. polymorphus*)
	1,787–2,338	**21–24**	6–8	7	34	**17**	*58*	24	29	15–20 × 9–18	Shen (1990)
	980–2,500	*12–20*	*9–17*	7	37	**21**	*56*	22	20	17–22 × 11–13.	Al-Zubaidy (2011)
*B. xiamenensis* Liu, 1994	1,620–2,180	*20–22*	9–11	7	**14**	**9**	**46**	*30*	*34*	18–22 × 12–16	Liu (1994)
*B. yamagutii* Gupta & Singh, 1985	1,460–1,570	*16*	8	*5*	*28*	*29*	66	24–25	30	15–18 × 9–11	Gupta & Singh (1985)

^a^This species is unrecognisable; the illustrations are poorly reproduced microphotographs

## Concluding remarks

It is not clear why we have not been successful in securing useful DNA from this species as it was fixed in the same way as other digeneans recovered from the Bali Workshop that have been successfully sequenced (Cribb et al., 2014; Bray et al., 2016; Yong et al., 2016; Bray et al., 2017). Successful and experienced molecular biologists in the Rostock and the University of Queensland Laboratories have been frustrated in their attempts to extract DNA from these worms. The species described here is morphologically distinct enough to be easily recognised so it was felt worthwhile to describe it and add a further detail to our depauperate knowledge of the marine fish digeneans of Indonesia.

We are aware of eleven named species of bucephalids in Indonesian waters. These are:

- *Bucephalus damriyasai* n. sp. ex blacktip trevally *Caranx heberi* (Carangidae), off Bali.
- *Bucephalus margaritae* Ozaki & Ishibashi, 1934 (as *B*. *retractilis* Yamaguti, 1952) ex *Caranx* sp. (Carangidae), off Sulawesi (Yamaguti, 1952).
- *Bucephalus sphyraenae* Yamaguti, 1952 ex *Sphyraena* sp. (Sphyraenidae), off Sulawesi (Yamaguti, 1952).
- *Prosorhynchoides tenuis* (Yamaguti, 1952) ex Indian flathead *Platycephalus indicus* (L.) (Platycephalidae), off Sulawesi (Yamaguti, 1952).
- *Prosorhynchus chorinemi* Yamaguti, 1952 ex doublespotted queenfish *Scomberoides lysan* (Forsskål) (Carangidae), off Sulawesi (Yamaguti, 1952).
- *Prosorhynchus longicollis* Yamaguti, 1953 ex *Sphyraena* sp. (Sphyraenidae), off Sulawesi (Yamaguti, 1953).
- *Prosorhynchus luzonicus* Velasquez, 1959 ex orange-spotted grouper *Epinephelus coioides* (Hamilton, 1822) (Serranidae), and brown-marbled grouper *E. fuscoguttatus* (Forsskål), off Sumatra and Java (Palm & Rückert, 2009; Rückert et al., 2009; Rückert et al., 2010; Kleinertz & Palm, 2015).
- *Prosorhynchus platycephali* (Yamaguti, 1934) ex fringelip flathead *Sunagocia otaitensis* (Cuvier) (Platycephalidae), off Java (Bray & Palm, 2009).
- *Rhipidocotyle danai* Bray & Palm, 2009 ex black snoek *Thyrsitoides marleyi* Fowler (Gempylidae), off Java (Bray & Palm, 2009).
- *Rhipidocotyle jayai* Bray & Palm, 2009 ex largefin croaker *Johnius macropterus* (Bleeker) (Sciaenidae), off Java (Bray & Palm, 2009).
- *Rhipidocotyle khalili* Nagaty, 1937 ex *Sphyraena* sp. (Sphyraenidae), off Sulawesi (Yamaguti, 1953).

Two further, as yet unnamed, species are found in Indonesian groupers:

- *Prosorhynchus* sp. 1 of Bray & Palm (2009) (syn. *Prosorhynchus australis* of Rückert et al. (2009) and Palm & Rückert (2009)) from *Epinephelus coioides*, off Sumatra, *E. fuscoguttatus*, off Java and the areolate grouper *E. areolatus* (Forsskål), off Java (Palm & Rückert, 2009; Rückert et al., 2009; Rückert et al., 2010; Palm et al., 2011; Kleinertz et al., 2014; Kleinertz & Palm, 2015).
- *Prosorhynchus* sp. 2 of Bray & Palm (2009) (Syn. *Prosorhynchus* cf. *crucibulum* (Rudolphi, 1819) of Palm & Rückert (2009)) ex *Epinephelus fuscoguttatus*, off Java and *E. areolatus*, off Bali (Rückert et al., 2009; Palm et al., 2011; Kleinertz et al., 2014).

## References

[CR1] Al-Zubaidy AB (2011). Digenetic trematodes (Bucephalidae: *Bucephalus* Baer, 1827 and *Rhipidocotyle* Diesing, 1858) from Red Sea fishes, Yemen Coast. Journal of King Abdulaziz University - Marine Sciences.

[CR2] Al Kawari KSR, Saoud MFA, Ramadan MM (1996). Biodiversity of helminth parasites of fishes in the Arabian Gulf, with special reference to digenetic trematodes and cestodes. Qatar University Science Journal.

[CR3] Bartoli P, Bray RA, Gibson DI (2005). Three poorly known and rarely reported bucephalid species (Digenea) in fishes from the Western Mediterranean. Systematic Parasitology.

[CR4] Bilqees FM, Khatoon N, Haseeb MF (2006). *Bucephalus hexalobatus* n. sp. (Gasterostomata Odhner, 1905: Bucephalidae Poche, 1907: Bucephalinae Nicoll, 1914 [*sic*]) from the fish *Pomadasys olivaceus* of Karachi coast. International Journal of Biology and Biotechnology.

[CR5] Brahim Tazi NA, Meddour A, Nadjadi Z, Boutiba Z (2016). First records of helminth parasites of *Dicentrarchus labrax* in the Western Coast of Algeria. Journal of Applied Environmental Biological Sciences.

[CR6] Bravo-Hollis M, Sogandares-Bernal F (1956). Trematodes of marine fishes of Mexican waters IX. Four gasterostomes from the Pacific coast. Journal of Parasitology.

[CR7] Bray RA (1984). Some helminth parasites of marine fishes and cephalopods of South Africa: Aspidogastrea and the digenean families Bucephalidae, Haplosplanchnidae, Mesometridae and Fellodistomidae. Journal of Natural History.

[CR8] Bray RA, Cribb TH, Littlewood DTJ, Waeschenbach A (2016). The molecular phylogeny of the digenean family Opecoelidae Ozaki, 1925 and the value of morphological characters, with the erection of a new subfamily. Folia Parasitologica.

[CR9] Bray RA, Palm HW (2009). Bucephalids (Digenea: Bucephalidae) from marine fishes off the south-western coast of Java, Indonesia, including the description of two new species of *Rhipidocotyle* and comments on the marine fish digenean fauna of Indonesia. Zootaxa.

[CR10] Bray RA, Palm HW, Cutmore SC, Cribb TH (2017). Three members of *Opisthomonorcheides* Parukhin, 1966 (Digenea: Monorchiidae) from carangid fishes (Perciformes) from Indonesia, with a review of the genus. Systematic Parasitology.

[CR11] Chinchilla OL, Mago Y, Fuentes JL (2006). Hallazgo de *Bucephalus margaritae* Ozaki et Ishibashi, 1934 (Trematoda: Bucephalidae) en ejemplares de *Sphyraena picudilla* Poey, 1860 (Sphyraenidae) capturados en la Bahia de Mochima, estado Sucre, Venezuela. Boletin del Instituto Oceanografico de Venezuela Universidad de Oriente.

[CR12] Corkum KC (1967). Bucephalidae (Trematoda) in fishes of the northern Gulf of Mexico: *Bucephalus* Baer, 1827. Transactions of the American Microscopical Society.

[CR13] Cribb TH, Bray RA (2010). Gut wash, body soak, blender, and heat-fixation: Approaches to the effective collection, fixation and preservation of trematodes of fishes. Systematic Parasitology.

[CR14] Cribb TH, Miller TL, Bray RA, Cutmore SC (2014). The sexual adult of *Cercaria praecox* Walker, 1971 (Digenea: Fellodistomidae), with the proposal of *Oceroma* n. g. Systematic Parasitology.

[CR15] Culurgioni J, Figus V, Cabiddu S, Murtas RD, Cau A, Sabatini A (2015). Larval helminth parasites of fishes and shellfishes from Santa Gilla Lagoon (Sardinia, Western Mediterranean), and their use as bioecological indicators. Estuaries and Coasts.

[CR16] Culurgioni J, Murtas RD, Cannella S, Figus V (2010). Parasites of wild European sea bass *Dicentrarchus labrax* (Linnaeus, 1758) from St. Gilla lagoon (Sardinia, South-western Mediterranean). Ittiopatologia.

[CR17] Culurgioni J, Sabatini A, De Murtas R, Mattiucci S, Figus V (2014). Helminth parasites of fish and shellfish from the Santa Gilla Lagoon in southern Sardinia, Italy. Journal of Helminthology.

[CR18] Dwivedi DK (2007). *Bucephalus arabiansis* sp. nov. (Trematoda: Bucephalidae) from a marine fish from Cochin coast, Kerala. Journal of Applied Bioscience.

[CR19] Dyer WG, Williams EH, Williams LB (1988). Digenetic trematodes of marine fishes of Okinawa, Japan. Journal of Parasitology.

[CR20] Eckmann F (1932). Beiträge zur Kenntnis der Trematodenfamilie Bucephalidae. Zeitschrift für Parasitenkunde.

[CR21] El-Naffar MKI, Gobashy A, El-Etreby SC, Kardousha MM (1992). General survey of helminth parasite genera of Arabian Gulf fishes (coasts of United Arab Emirates). Arab Gulf Journal of Scientific Research.

[CR22] Fischthal JH (1982). Additional records of digenetic trematodes of marine fishes from Israel’s Mediterranean coast. Proceedings of the Helminthological Society of Washington.

[CR23] Fischthal JH, Carson DO, Vaught RS (1982). Comparative allometry of size of the digenetic trematode *Bucephalus gorgon* (Linton, 1905) Eckmann, 1932 (Bucephalidae) in two sites of infection in the marine fish *Seriola dumerili* (Risso). Journal of Parasitology.

[CR24] Gargouri Ben Abdallah L, Maamouri F (2002). Cycle évolutif de *Bucephalus anguillae* Spakulová, Macko, Berrilli & Dezfuli, 2002 (Digenea, Bucephalidae) parasite de *Anguilla anguilla* (L.). Systematic Parasitology.

[CR25] Gargouri Ben Abdallah L, Maamouri F (2005). The life cycle of *Bucephalus labracis* Paggi and Orecchia, 1965 (Digenea, Bucephalidae), a parasite of *Dicentrarchus labrax* in Tunisia. Bulletin of the European Association of Fish Pathologists.

[CR26] Gijon-Botella H, Medina M, Lopez-Roman R (2007). Aportacion al catalogo de Bucephaloidea Poche, 1907 de peces marinos del Archipielago de Canarias. Research and Reviews in Parasitology.

[CR27] Gupta PC, Singh RB (1985). Four new digenetic trematodes from marine fishes off Puri coast, Bay of Bengal. Indian Journal of Parasitology.

[CR28] Gupta V, Tiwari M (1985). Trematode parasites of marine fishes. Indian Journal of Helminthology.

[CR29] Hopkins SH (1956). Two new trematodes from Louisiana, and the excretory system of Bucephalidae. Transactions of the American Microscopical Society.

[CR30] Hutson KS, Ernst I, Mooney AJ, Whittington ID (2007). Metazoan parasite assemblages of wild *Seriola lalandi* (Carangidae) from eastern and southern Australia. Parasitology International.

[CR31] ICZN (2012). *International Commission on Zoological Nomenclature*: Amendment of articles 8, 9, 10, 21 and 78 of the International Code of Zoological Nomenclature to expand and refine methods of publication. Bulletin of Zoological Nomenclature.

[CR32] Kleinertz S, Damriyasa IM, Hagen W, Theisen S, Palm HW (2014). An environmental assessment of the parasite fauna of the reef-associated grouper *Epinephelus areolatus* from Indonesian waters. Journal of Helminthology.

[CR33] Kleinertz S, Palm HW (2015). Parasites of the grouper fish *Epinephelus coioides* (Serranidae) as potential environmental indicators in Indonesian coastal ecosystems. Journal of Helminthology.

[CR34] Kohn A (1966). *Bucephalus solitarius* sp. n., parasito de peixe do litoral brasileiro (Trematoda, Bucephaliformes). Atas da Sociedade de Biologia do Rio de Janeiro.

[CR35] Linton E (1905). Parasites of fishes of Beaufort, North Carolina. Bulletin of the Bureau of Fisheries for 1904.

[CR36] Linton E (1940). Trematodes from fishes mainly from the Woods Hole region, Massachusetts. Proceedings of the United States National Museum.

[CR37] Liu S (1994). Report on Bucephalidae trematodes with description of one new species from coastal areas of Fujian, China. Zoological Research.

[CR38] Luque J, Oliva M (1993). Trematodes of marine fishes from the Peruvian Faunistic Province (Peru and Chile), with description of *Lecithochirium callaoensis* n. sp. and new records. Revista de Biologia Marina.

[CR39] Maillard C (1975). *Labratrema lamirandi* (Carrère, 1937) (Trematoda, Bucephalidae) parasite du *Dicentrarchus labrax* (L., 1758). Création du genre *Labratrema*. Cycle évolutif. Bulletin du Muséum National d’Histoire Naturelle.

[CR40] Maillard, C. (1976). *Distomatoses de poissons en milieu lagunaire*. PhD thesis, Académie de Montpellier, Université des Sciences et Techniques du Languedoc, Montpellier.

[CR41] Maillard C, Saad-Fares A (1981). *Bucephalus baeri* n. sp. (Trematoda) parasite de *Dicentrarchus labrax* (Teleostei): Description et cycle évolutif. Zeitschrift für Parasitenkunde.

[CR42] Manter HW (1934). Some digenetic trematodes from deep-water fish of Tortugas, Florida. Papers from Tortugas Laboratory.

[CR43] Manter HW (1940). Digenetic trematodes of fishes from the Galapagos Islands and the neighboring Pacific. Allan Hancock Pacific Expeditions.

[CR44] Manter HW (1940). Gasterostomes (Trematoda) of Tortugas, Florida. Papers from the Tortugas Laboratory of the Carnegie Institute of Washington.

[CR45] Marchiori N d C, Magalhães ARM, Pereira J (2010). The life cycle of *Bucephalus margaritae* Ozaki & Ishibashi, 1934 (Digenea, Bucephalidae) from the coast of Santa Catarina State, Brazil. Acta Scientiarum. Biological Sciences.

[CR46] Montero, F. E., Aznar, F. J., Fernandez, M., & Raga, J. A. (2002). Parasite fauna in cultured greater amberjacks (*Seriola dumerili*) from the Spanish Mediterranean. In: *Tenth International Congress of Parasitology, Vancouver*, 2002, p. 203.

[CR47] Muñoz MV, Fernandez JP, Carbonell E, Orts ME (1989). Contribucion al estudio de algunos bucefalidos (Trematoda: Bucephalidae) parasitos de peces marinos de aguas ibericas. Revista Ibérica de Parasitología.

[CR48] Nagaty, H. F. (1937). *Trematodes of fishes from the Red Sea. Part 1. Studies on the family Bucephalidae Poche, 1907* (Vol. Faculty of Medicine, Publication No. 12). Cairo: Egyptian University, 172 pp.

[CR49] Nahhas FM, Powell EC (1971). Digenetic trematodes of marine fishes from the Floridian northern Gulf of Mexico. Tulane Studies in Zoology and Botany.

[CR50] Nahhas FM, Sey O, Nakahara G (2006). Digenetic trematodes of marine fishes from the Arabian Gulf off the coast of Kuwait. Family Bucephalidae Poche, 1907, and the description of a new species. Helminthologia.

[CR51] Neubert K, Yulianto I, Kleinertz S, Theisen S, Wiryawan B, Palm HW (2016). Parasite fauna of white-streaked grouper, *Epinephelus ongus* (Bloch, 1790) (Epinephelidae) from Karimunjawa, Indonesia. Parasitology Open.

[CR52] Nisreen Ezz El-Dien M, Abdel-Rahman MS, El-Gawady HMA, Imam EAE, Fahmy MM (1990). Detection of two new trematode species from marine fishes in Egypt. Journal of the Egyptian Veterinary Medical Association.

[CR53] Nolan MJ, Curran SS, Miller TL, Cutmore SC, Cantacessi C, Cribb TH (2015). *Dollfustrema durum* n. sp. and *Heterobucephalopsis perardua* n. sp. (Digenea: Bucephalidae) from the giant moray eel, *Gymnothorax javanicus* (Bleeker) (Anguilliformes: Muraenidae), and proposal of the Heterobucephalopsinae n. subfam. Parasitology International.

[CR54] Oshmarin, P. G. (1965). [On the trematode fauna of marine and freshwater fishes of Vietnam]. In: Leonov, A. A., Mamaev, Y. L., & Oshmarin, P. G. (Eds), *Parasitic worms of domestic and wild animals*). Vladivostok: Akademiya Nauk SSSR, pp. 213–249 (In Russian).

[CR55] Overstreet RM (1969). Digenetic trematodes of marine teleost fishes from Biscayne Bay, Florida. Tulane Studies in Zoology and Botany.

[CR56] Ozaki Y, Ishibashi C (1934). Notes on the cercaria of pearl oyster. Proceedings of the Imperial Academy.

[CR57] Paggi L, Orecchia P (1965). Su un nuovo trematode parassita dell’intestino di *Morone labrax*: *Bucephalus labracis* n. sp. Parassitologia.

[CR58] Palm, H. W. (2011). Fish parasites as biological indicators in a changing world: Can we monitor environmental impact and climate change? In: Mehlhorn H. (Ed.), *Progress in Parasitology Parasitology Research Monographs, Vol. 2*. Berlin: Springer, pp. 223–250.

[CR59] Palm HW, Bray RA (2014). Marine Fish Parasitology in Hawaii.

[CR60] Palm HW, Kleinertz S, Rückert S (2011). Parasite diversity as an indicator of environmental change? An example from tropical grouper (*Epinephelus fuscoguttatus*) mariculture in Indonesia. Parasitology.

[CR61] Palm HW, Rückert S (2009). A new approach to visualize ecosystem health by using parasites. Parasitology Research.

[CR62] Parukhin, A. M. (1966). [Helminth fauna of carangid fish from the South China Sea]. *Biologiya Morya, Kiev, vol,* 80–96 (In Russian).

[CR63] Parukhin AM (1970). On the study of trematode fauna in fish from the Red Sea and Aden Bay. Biologiya Morya, Kiev.

[CR64] Parukhin, A. M. (1976). [*Parasitic worms of food fishes of the southern Seas*]. Kiev: Naukova Dumka, 183 pp (In Russian).

[CR65] Parukhin, A. M. (1979). [New species of trematodes of fishes from the Indian Ocean and Red Sea.] *Parazitologiya, 13*, 639–643 (In Russian). 514634

[CR66] Pina S, Barandela T, Santos MJ, Russell-Pinto F, Rodrigues P (2009). Identification and description of *Bucephalus minimus* (Digenea: Bucephalidae) life cycle in Portugal: morphological, histopathological, and molecular data. Journal of Parasitology.

[CR67] Proctor, S. P., Merta, I. G. S., Sondita, M. F. A., Wahju, R. I., Davis, T. L. O., Gunn, J. S., & Andamari, R. (2003). A review of Indonesia’s Indian Ocean Tuna Fisheries. ACIAR Project FIS/2001/079. 106 pp.

[CR68] Rückert S, Klimpel S, Al-Quraishy S, Mehlhorn H, Palm HW (2009). Transmission of fish parasites into grouper mariculture (Serranidae: *Epinephelus coioides* (Hamilton, 1822)) in Lampung Bay, Indonesia. Parasitology Research.

[CR69] Rückert S, Klimpel S, Palm HW (2010). Parasites of cultured and wild brown-marbled grouper *Epinephelus fuscoguttatus* (Forsskal, 1775) in Lampung Bay, Indonesia. Aquaculture Research.

[CR70] Sakaguchi S (1962). Studies on a Trematoda parasitic on pearl oyster. I. On the encystation of cercaria, *Bucephalus margaritae*. Bulletin of the National Pearl Research Laboratory.

[CR71] Sakaguchi S (1964). Studies on a trematode parasite on pearl oyster. II. Its effects on pearl oyster as the first intermediate host. Bulletin of the National Pearl Research Laboratory.

[CR72] Sakaguchi S (1966). Studies on a trematode parasite of the pearl oyster. III. The metacercaria obtained from the second intermediate host artificially infected with the cercaria, *Bucephalus margaritae*. Bulletin of the Japanese Society of Scientific Fisheries.

[CR73] Sakaguchi S (1966). Studies on a trematode parasite of the pearl oyster. IV On the Trematoda of genus *Bucephalus* found in the fishes, *Caranx sexfasciatus* and *C. ignobilis*. Bulletin of the Japanese Society of Scientific Fisheries.

[CR74] Sakaguchi S (1968). Studies on the life-history of the trematode parasitic in pearl oyster, *Pinctada fucata,* and on the hindrance for pearl culture. Bulletin of the National Pearl Research Laboratory.

[CR75] Shen, J.-w. (1990). *Digenetic trematodes of marine fishes from Hainan Island*. Beijing: Science Publications, 228 pp (In Chinese, English summary).

[CR76] Špakulová M, Macko JK, Berrilli F, Dezfuli BS (2002). Description of *Bucephalus anguillae* n. sp (Trematoda : Bucephalidae), a parasite of the Eel *Anguilla anguilla* (Anguillidae) from a brackish water lagoon of the Adriatic Sea. Journal of Parasitology.

[CR77] Srivastava HD (1938). Studies on the gasterostomatous parasites of Indian food fishes. Indian Journal of Veterinary Science and Animal Husbandry.

[CR78] Stossich M (1887). Brani di elmintologia tergestina. Serie quarta. Bollettino della Società Adriatica di Scienze Naturali in Trieste.

[CR79] Szidat L (1961). Versuch einer Zoogeographie des Sud-Atlantik mit Hilfe von Zeitparasiten der Meeresfische. Parasitologische Schriftenreihe.

[CR80] Truong TV, Neubert K, Unger P, Bui TQ, Ngo HTT, Palm HW, Kleinertz S (2017). Assessment of *Epinephelus coioides* (Hamilton, 1822) aquaculture systems in the Gulf of Tonkin, Vietnam, by using fish parasites. Journal of Applied Ichthyology.

[CR81] Velasquez CC (1959). Studies on the family Bucephalidae Poche 1907 (Trematoda) from Philippine food fishes. Journal of Parasitology.

[CR82] Velasquez, C. C. (1975). *Digenetic trematodes of Philippine fishes*. Quezon: University of the Philippines Press, 140 pp.

[CR83] Verma SC (1936). Studies on the family Bucephalidae (Gasterostomata), Part II. Descriptions of two new forms from Indian marine fishes. Proceedings of the National Academy of Sciences, India.

[CR84] Vlasenko P (1931). [On the parasitic worm fauna of fishes of the Black Sea]. *Trudy Karadahs’koyi Nauchnoyi Stantsiyi imeni T.I*. Vyazems’koho.

[CR85] Wang P-Q (1977). A survey of the parasites of eels from Fujian Province, China, II. Journal of Fujian Teachers University (Natural Science).

[CR86] Wang P-Q (1980). Studies on some species of gasterostome trematodes from Fujian, China. Acta Zootaxonomica Sinica.

[CR87] Yadav BB (1977). *Bucephalus elacatus* n. sp. (Trematoda: Bucephalidae) from the marine fish, *Elacate nigra* (Gunther), in India. Marathwada University Journal of Science (Biol. Sci.).

[CR100] Yamaguti S (1934). Studies on the helminth fauna of Japan. Part 2. Trematodes of fishes, I. Japanese Journal of Zoology.

[CR88] Yamaguti S (1952). Parasitic worms mainly from Celebes. Part 1. New digenetic trematodes of fishes. Acta Medicinae Okayama.

[CR89] Yamaguti S (1953). Parasitic worms mainly from Celebes. Part 3. Digenetic trematodes of fishes. II. Acta Medicinae Okayama.

[CR90] Yamaguti S (1959). Studies on the helminth fauna of Japan. Part 54. Trematodes of fishes, XII. Publications of the Seto Marine Biological Laboratory.

[CR91] Yamaguti, S. (1970). *Digenetic trematodes of Hawaiian fishes*. Tokyo: Keigaku, 436 pp.

[CR92] Yong RQY, Cutmore SC, Bray RA, Miller TL, Semarariana IWY, Palm HW, Cribb TH (2016). Three new species of blood flukes (Digenea: Aporocotylidae) infecting pufferfishes (Teleostei: Tetraodontidae) from off Bali, Indonesia. Parasitology International.

